# A29 HIGH DIMENSIONAL SINGLE CELL ANALYSIS OF IL-4 TREATED MACROPHAGES REVEALS A HETEROGENIC PHENOTYPE AND THE ABSENCE OF CD206+ SUBSETS IN CROHN'S DISEASE PATIENTS

**DOI:** 10.1093/jcag/gwac036.029

**Published:** 2023-03-07

**Authors:** B E Callejas, K Flanigan, J Schlechte, J A Sousa, A Wang, B McDonald, R Paniconne, D M McKay

**Affiliations:** 1 Gastrointestinal Research Group, Inflammation Research Network, Department of Physiology and Pharmacology, The Calvin Phoebe and Joan Snyder Institute for Chronic Diseases, University of Calgary; 2 Division of Gastroenterology and Hepatology, Department of Medicine, University of Calgary, Calgary, Canada

## Abstract

**Background:**

Individuals with IBD have a reduced quality of life and while treatment has improved, a cure remains elusive. Interleukin-4 differentiated macrophages (M(IL4)s) from healthy donors and IBD patients in remission promote epithelial wound repair and reduce the severity of colitis in a mouse model: findings that support the autologous transfer of M(IL4)s to treat IBD. However, issues remain if this novel therapy is to advance to clinical evaluation: a much greater understanding of the possible heterogeneity within the human M(IL4) population, the biology of any sub-groups therein and the biology of any M(IL4) subtypes is needed.

**Purpose:**

To determine if cryopreservation, sex, and disease status in Crohn's disease (CD) impact the phenotype and function of M(IL4)

**Method:**

Blood mononuclear cells were collected from healthy donors, and individuals with active CD or in remission (n=6/group), and differentiated to macrophages with GMP-grade recombinant human M-CSF (10 ng/ml, 7 days). Macrophages (2.5 x10^5^) were activated with IL-4 (10ng/ml, 48h). Fresh and cryopreserved M(0) and M(IL4) were stained using a panel of 18 metal-conjugated antibodies. Data were acquired on a Helios CyTOFII mass cytometer. FCS files were imported into Cytobank for manual gating (viable CD45^+^CD3^-^CD19^-^ singlet cell events) and exported for meta-clustering in R with the CATALYST package using the FlowSOM function with 10 metaclusters.

**Result(s):**

FlowSOM analysis on M(IL4) from healthy donors indicated clear differences between M(0) and M(IL4), with 8 metaclusters >1.5% of M(IL4)s: the most abundant metaclusters express high and intermediate CD206, PDL1, CD11b, CD33, CD64, HLA-DR and low CD14 levels. There were no significant differences in these M(IL4)s metaclusters between males and females, or fresh vs. cryopreserved cells using this characterization strategy. M(IL4) from patients with active CD, were markedly different with the absence of three high-intermediate CD206^+^PDL1^+^ metaclusters and a general lack of CD206^+^ metaclusters compared to healthy controls. Surprisingly, and despite M(IL4)s from CD patients in remission showing increased CD206 mRNA, CYTOF analysis revealed that the macrophages were more similar to those from individuals with active disease than those from healthy controls

**Conclusion(s):**

Here human macrophages activated with IL4 *in vitro* are revealed as a heterogeneous population, and subsets characterized by increased high CD206 expression are generally absent in active Crohn’s disease. Intriguing while prior experiments with M(IL4) from CD patients in remission reduced the severity of colitis in *rag1*^*-/*-^ mice, the characterization of these cells via 18 protein markers indicates they are more like M(IL4)s from patients with active disease compared to healthy controls. While presenting autologous M(IL4) transfer as a novel approach for IBD, a much fuller understanding of the phenotype and function of M(IL4)s (and sub-populations therein) is required before this would become a therapeutic option

**Please acknowledge all funding agencies by checking the applicable boxes below:**

CAG

**Disclosure of Interest:**

None Declared

